# Supplementary material for: Conflict detection and resolution in macaque frontal eye fields
Source: Commun Biol. 2024 Jan 23;7:119. doi: 10.1038/s42003-024-05800-x (PMC10805886; doi:10.1038/s42003-024-05800-x)
Supplement: Supplementary file 2 — Supplementary Information [file 42003_2024_5800_MOESM2_ESM.pdf]

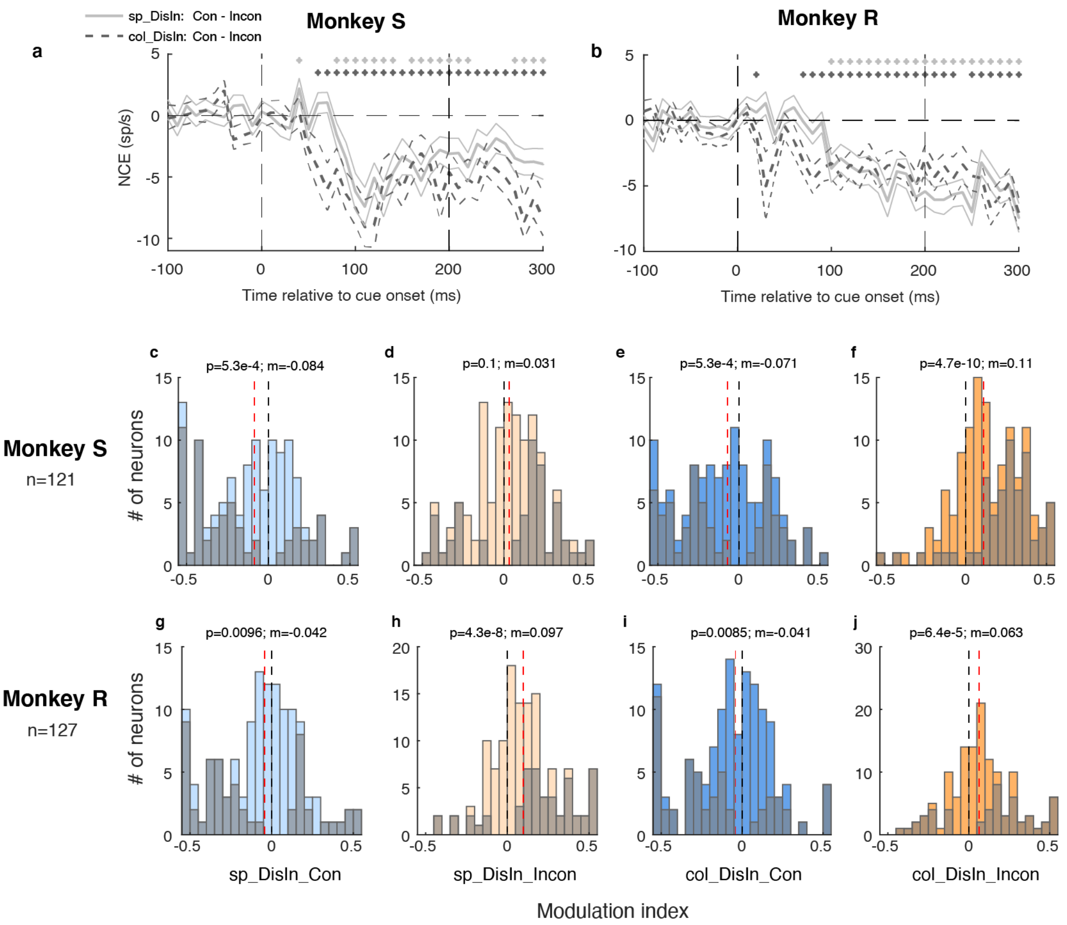

Supplementary Figure 1. Time-course of the NCE for distractor encoding neurons under two rules. *(**a, b**)The average response differences between congruent and incongruent conditions (i.e., the NCE) for the two monkeys. The thin lines indicate the s.e.m. across neurons. The stars indicate that the NCEs are significantly different from zero for a given moving 50ms (stepped by 10ms) time-bin (two-tailed WSRT, p<0.05): grey and black stars for spatial and color rules, respectively. The time is relative to cue onset (= 0ms), the PSTHs are not smoothed, and the two vertical dashed lines indicate cue onset and offset. (**c-j**) The response changes after the cue for distractor-encoding neurons for both the spatial and color rules for the two monkeys. The modulation index (MI) was calculated as the difference between the average response (100-300ms after the cue onset) and the baseline (200ms preceding the cue) divided by their sum. The black vertical lines indicate zero change, and the red vertical lines indicate the medians for each condition. Negative MIs indicate suppressed responses after the cue compared to the baseline, while positive MIs indicate increased responses. The final bars on the two sides of the histogram sum all data values beyond -0.5 or 0.5. The p values (WSRT) indicate whether the medians of the MIs are significantly different from zero. The colors of the bars are matched in Figure 3a. The gray bars indicate the number of neurons showing significant effects (p<0.05, two-tailed WSRT).*

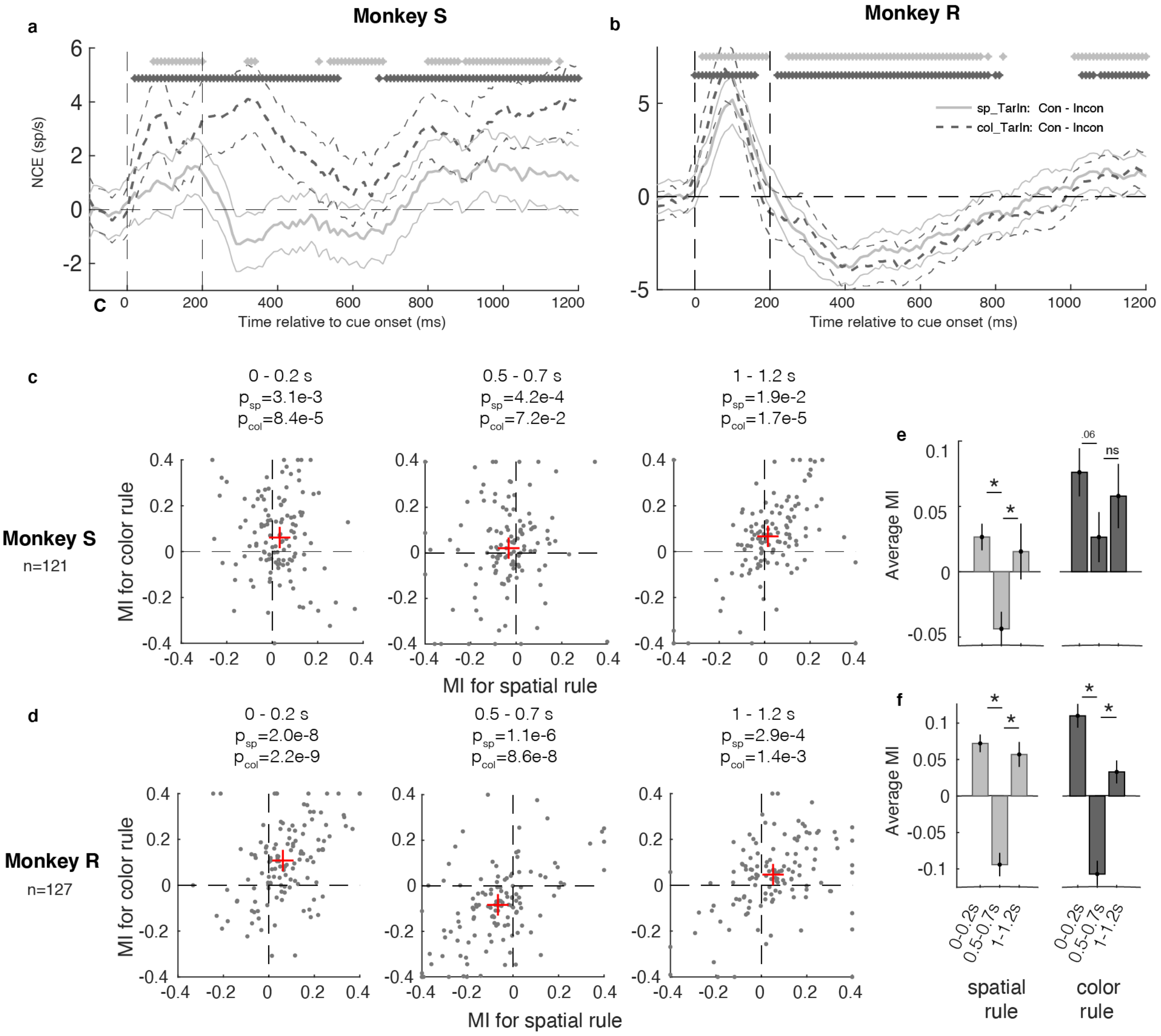

Supplementary Figure 2. Time-course of the NCE for target encoding neurons under two rules. *The legend is the same as Figure 4b-d for the two monkeys separately. (**a**, **b**) The NCE for target-encoding neurons under two rules for two monkeys. The thin lines indicate the s.e.m. across neurons. The stars indicate that the NCEs are significantly different from zero for a given moving 50ms (stepped by 10ms) time-bin (two-tailed WSRT, p<0.05): grey and black stars for spatial and color rules, respectively. (**c**) Same as b, but NCEs for the color and spatial trails focus on a window between -100 and 300ms relative to cue onset (= 0ms), and PSTHs are not smoothed. The two vertical dashed lines indicate cue onset and offset in A-C. (**c, d**) Scatter plots of MIs for color rule (y-axis) and spatial rule (x-axis) trials in time bins of 0-0.2s (left), 0.5-0.7s (middle), and 1-1.2s (right) after cue onset. The red + indicates the median MIs. The p values (WSRT) show that the medians of the MIs are significantly different from zero for color (p_{col}) and spatial (p_{sp}) rule trials. $MI=(R_{con}-R_{incon})/(R_{con}+R_{incon})$. (**e, f**) The average MIs in 0-0.2s and 1-1.2s bins are significantly higher than the MI in the 0.5-0.7s bin for both spatial (left) and color (right) trials for two monkeys. The error bars indicate the s.e.m. across neurons. * : p<0.05, ns: not significant (2-tailed paired t-test).*

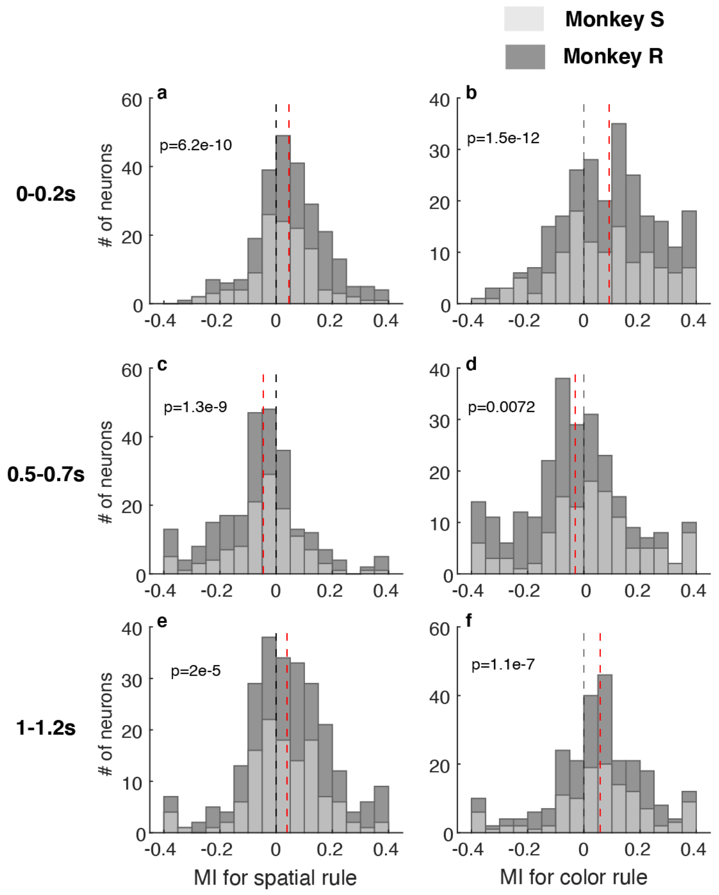

Supplementary Figure 3. The NCE in different time windows for target-encoding neurons under two rules. *The NCE for each neuron was indicated by the modulation index (MI), which was calculated as the average response difference of the congruent and incongruent trials divided by their sum, i.e., MI=(R_{con}-R_{incon})/(R_{con}+R_{incon}). The rows indicate the 3 time windows after the cue onset. The left and right columns are for the spatial (**a, c, e**) and color (**b, d, f**) rule trials. The black vertical lines indicate zero NCE, and the red vertical dashed lines indicate the medians for each time window. The final bars on the two sides of the histogram sum all data values beyond -0.4 or 0.4. The p values (WSRT) indicate whether the medians of the MIs are significantly different from zero.*

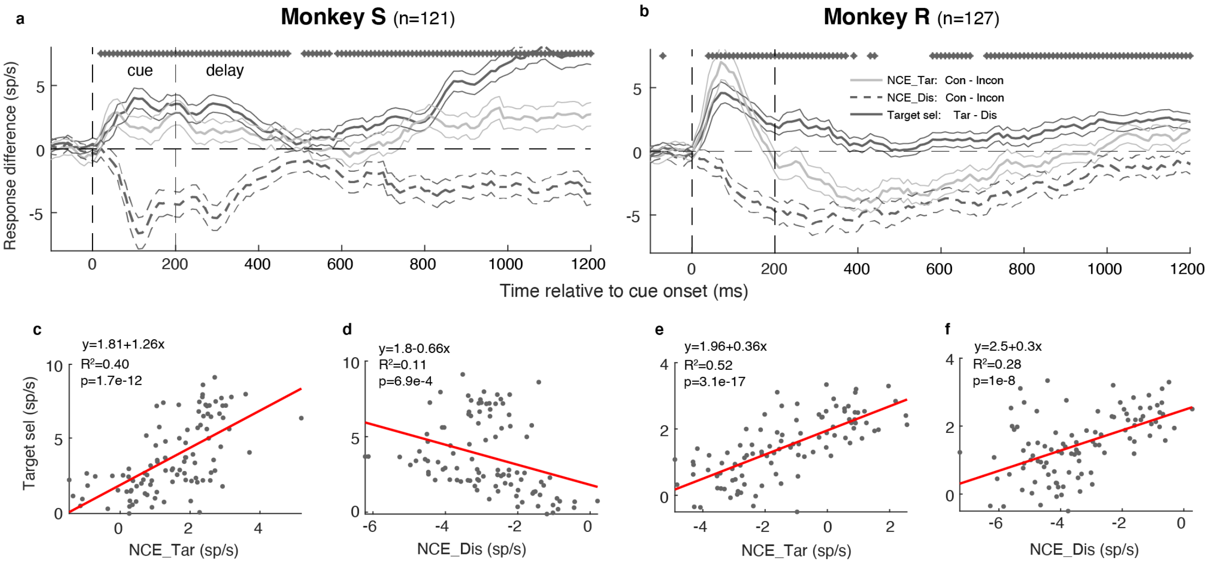

Supplementary Figure 4. Time-course of the NCE and overall target selection signal. ***(a, b)*** *The time-course of the average attention effect (black line), and the NCE of target (grey solid line) and distractor-encoding neurons (dashed lines) for the two monkeys. The thinner lines indicate the s.e.m across neurons. The stars indicate that the target selection signal is significantly different from zero for the given moving 50ms (stepped by 10ms) time bin.* ***(c-f)*** *The linear correlation between the target selection signal (y-axis) and the NCE (x-axis) of target- (****c, e****) and distractor-encoding neurons (****d, f****). The dots represent the average NCE and target attention signal in a 10ms non-smoothed time bin from 200 to 1200ms after cue onset. The linear regression lines (red), the model functions,* R^2*, and p values are shown.*
